# Development and In Vitro Evaluation of Liposomes Using Soy Lecithin to Encapsulate Paclitaxel

**DOI:** 10.1155/2017/8234712

**Published:** 2017-02-26

**Authors:** Thi Lan Nguyen, Thi Hiep Nguyen, Dai Hai Nguyen

**Affiliations:** ^1^Institute of Applied Materials Science, Vietnam Academy of Science and Technology, 01 TL29, District 12, Ho Chi Minh City, Vietnam; ^2^Can Tho University, 3/2 Street, Ninh Kieu District, Can Tho City, Vietnam; ^3^Tissue Engineering and Regenerative Medicine Group, Department of Biomedical Engineering, International University, Vietnam National University-HCMC (VNU-HCMC), Ho Chi Minh City 70000, Vietnam

## Abstract

The formulation of a potential delivery system based on liposomes (Lips) formulated from soy lecithin (SL) for paclitaxel (PTX) was achieved (PTX-Lips). At first, PTX-Lips were prepared by thin film method using SL and cholesterol and then were characterized for their physiochemical properties (particle size, polydispersity index, zeta potential, and morphology). The results indicated that PTX-Lips were spherical in shape with a dynamic light scattering (DLS) particle size of 131 ± 30.5 nm. Besides, PTX was efficiently encapsulated in Lips, 94.5 ± 3.2% for drug loading efficiency, and slowly released up to 96 h, compared with free PTX. More importantly, cell proliferation kit I (MTT) assay data showed that Lips were biocompatible nanocarriers, and in addition the incorporation of PTX into Lips has been proven successful in reducing the toxicity of PTX. As a result, development of Lips using SL may offer a stable delivery system and promising properties for loading and sustained release of PTX in cancer therapy.

## 1. Introduction

Discovered in 1962, paclitaxel (PTX) is one of the most powerful anticancer drugs for various types of solid tumors, especially for breast cancer and advanced ovarian carcinoma. However, PTX has several disadvantages such as poor water solubility, high toxicity, and low bioavailability, which limit its potential clinical application [1–4]. Among approaches to overcome these drawbacks, drug delivery system is suggested to be a promising candidate owing to the knowledge that nanocarriers can efficiently control the pharmacokinetic characteristics of drugs [5, 6]. This method can deliver medication within desired therapeutic range to abnormal cells without affecting normal cells while maintaining the systemic levels of drugs [7–13].

Considering the variety of nanocarriers, liposomes (Lips), spherical vesicles consisting of at least one phospholipid bilayer, have been investigated as potential carriers for drug delivery applications due to their high biocompatibility, complete biodegradability, low toxicity, and ability to entrap both water- and lipid-soluble functional compounds and simplify specific drug delivery to tumor site. Furthermore, the stability of the functional components encapsulated in Lips can be increased, therefore maintaining their activities in environments that typically result in rapid degradation. In addition, Lips properties differ considerably with regard to lipid composition, particle size, surface charge, and the method of Lips preparation. The rigidity/fluidity and the charge of the bilayer were strongly influenced by the choice of bilayer components, for instance, saturated or unsaturated phospholipids from natural sources such as egg or soybean phosphatidylcholine [5, 14, 15]. Among these choices, the use of soy lecithin (SL), a naturally occurring phospholipid derived from soybeans, not only provides much more permeable Lips but also facilitates large-scale industrial production because of the reduction of production costs as compared with saturated phospholipids [16]. Several studies have been conducted on the benefits of using SL to obtain desired Lips. Madrigal-Carballo et al. prepared multilayered biopolymer-coated Lips formulated from SL as a novel system for ellagic acid delivery. They successfully achieved monodispered and stable spherical Lips with a diameter of 386.5 ± 25.9 nm and a surface charge of −30.66 ± 1.55 nm by thin film fabrication technique for the liposomal system coated with four biopolymer layers. These results indicated that the biopolymer-coated Lips offer good features for loading into their liposomal core and slow release of ellagic acid [17]. Additionally, in a study conducted by Mura and coworkers, Lips made from SL and alkyl polyglucosides (OrNS10) were formulated and characterized for the purpose of designing suitable drug delivery systems for their potential uses. The stability of Lips was also studied by checking average particle size and zeta potential value variation of different liposomal formulations during 4 weeks. The results showed that the addition of OrNS10 to SL has the ability to improve Lips stability [18].

**Figure 1:**
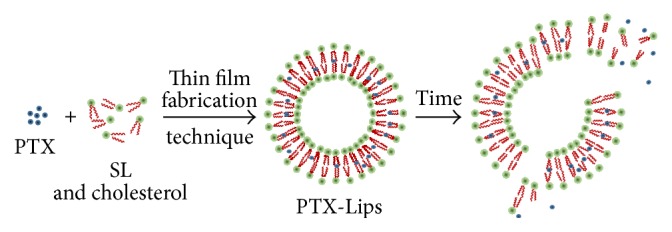
Schematic illustration of the formation of PTX-Lips and the release of PTX from Lips over time.

Herein, we developed Lips formulated from SL for PTX delivery (PTX-Lips). The formation of PTX-Lips was prepared according to the thin film method (Figure 1) and later these PTX-Lips were characterized by dynamic light scattering (DLS), zeta potential measurement, and transmission electron microscopy (TEM). Either drug loading or drug release behavior of PTX-Lips was also evaluated. Particularly, cell proliferation kit I (MTT) assay was used to determine the ability of PTX-Lips to minimize the toxicity to HeLa cells of PTX. This study is expected to improve the stability of Lips which was synthesized by eco-friendly SL for PTX delivery in cancer therapy.

## 2. Materials and Methods

### 2.1. Materials

PTX was supplied by Samyang Corporation (Seoul, Korea). Lecithin from soybean (CAS number 8002-43-5) and Tween 80 (polyoxyethylene sorbitan monooleate, CAS number 900 5-65-6) were purchased from Tokyo Chemistry Industry Co., Ltd. (Kita-ku, Tokyo, Japan). Cholesterol was obtained from Sigma-Aldrich (St Louis, MO, USA). Cetyltrimethylammonium bromide (CTAB) was purchased from Merck (Darmstadt, Germany). All chemicals and solvents were of highest analytical grade and used without further purification.

### 2.2. Methods

#### 2.2.1. Preparation of PTX-Lips

PTX-Lips were prepared by conventional thin film technique using SL and cholesterol. Briefly, SL (500 mg), cholesterol (56 mg), CTAB (5 mg), and 5% PTX (32 mg) were dissolved in chloroform-methanol (2 : 1 v/v) at room temperature. The mixture was evaporated in a rotary evaporator (Büchi Rotavapor R-114, Essen, Germany) at 45°C for 4 h, resulting in a formation of thin lipid film. The obtained thin films were hydrated with 15 mL of deionized water (deH_2_O) containing 80 mg of Tween 80 under constant stirring at 60°C. The suspension was further homogenized (EmulsiFlex-05 homogenizer, Avestin Inc., Ottawa, Canada) at 800 bar for 5 cycles, followed by centrifugation at 5500 rpm for 30 min to remove nonencapsulated PTX. The resulting sample was then lyophilized using 10% mannitol as cryoprotectants and stored at 2–8°C.

#### 2.2.2. Characterization

The particle size and polydispersity index of PTX-Lips were measured by DLS using a Zetasizer Nano ZS (ZEN 3600, Malvern Instruments Ltd., Malvern, Worcestershire, UK). A helium-neon (He-Ne) ion laser at 633 nm was used as the incident beam. The detection angle and the temperature were 90° and 25°C, respectively. All samples (1 mg/mL) were sonicated for 15 min, filtered (pore size = 0.45 *μ*m), and carried out at 37°C. The zeta potential of PTX-Lips was also measured using a Zetasizer Nano ZS ZEN 3600. All measurements were made in triplicate for each sample. The size and morphology of PTX-Lips were confirmed by TEM using JEM-1400 (300 kV; JEOL, Tokyo, Japan). The samples were prepared by placing a drop of solution in deH_2_O (1 mg/mL) onto a carbon-copper grid (300-mesh, Ted Pella, Inc., USA) and air-drying for 10 min.

#### 2.2.3. PTX Loading Contents and In Vitro PTX Release

In order to determine the PTX loading contents in Lips, PTX-Lips were first mixed with 1% Triton X100, incubated for 1 h, and centrifuged at 6000 rpm for 30 min at 25°C to separate PTX from Lips. The total PTX contents in Lips were measured using a Shimadzu LC-20A Prominence System (Shimadzu, Kyoto, Japan). The injected volume was 10 *μ*L and the mobile phase (acetonitrile : water = 50 : 50 v/v) was delivered at 1.00 mL/min. A reverse-phase Fortis C18 column (150 4.6 mm i.d., pore size 5 *μ*m; Fortis Technologies Ltd., Cheshire, UK) was used, and column effluent was monitored with a UV detector at 227 nm. The calibration curve for quantification of PTX in Lips was found to be linear over the standard PTX concentration range of 0–50,000 ng/mL with a high correlation coefficient of *R*^2^ = 0.998. The following equations were used to calculate the drug loading efficiency (DLE) and drug loading content (DLC):

(1) DLE%=weight  of  PTX  in  Lipsweight  of  PTX  feed  initially×100,DLC%=weight  of  PTX  inLipsweight  of  Lips  and  PTX×100.

The in vitro PTX release experiments were performed in PBS buffer (0.01 M, pH 7.4) at 37°C using dialysis method. Initially, 1 mL of PTX-Lips suspended in PBS containing 2% Tween 80 was transferred to a dialysis bag (MWCO 6–8 kDa, Spectrum Laboratories, Inc., Rancho Dominguez, CA, USA) and immersed into 20 mL of the release medium in vials at 37°C. The vials were then placed in an orbital shaker bath, which was maintained at 37°C and shaken horizontally at 100 rpm. At specific time intervals, 2 mL of the release medium was collected and an equal volume of fresh medium was added. The collected samples were filtered (pore size = 0.22 *μ*m) before high performance liquid chromatography analysis.

#### 2.2.4. MTT Viability Test

The MTT assay was used to evaluate cytotoxicity of PTX-Lips on Hela cells. The cells were seeded in a 96-well plate at a density of 1 × 10^4^ cells/well in 130 *μ*L of Dulbecco's Modified Eagle's medium (DMEM) supplemented with 10% FBS and 1% penicillin-streptomycin and cultured 1 day at 37°C. Then, the medium was removed and the cells were incubated with samples. The cells were incubated for 48 h, followed by removing medium and washing twice with PBS. MTT solution (25 *μ*L) and culture medium (130 *μ*L) were added to each well and the cells were cultured for 3 h. DMSO (130 *μ*L) was added to each well to dissolve the precipitate. The cells cultured with medium only were used as a control and assigned to 100% survival. The absorbance was measured at 570 nm using a multiplate reader (SpectraMax M2e, Molecular Devices Co., USA). Cell viability of all other groups was calculated by normalization of its absorbance intensity to that of “Ctrl” group with the following equation:

(2) Cell  viability%=Abssample−AbsblankAbscontrol−Absblank×100%.

## 3. Results and Discussion

### 3.1. Characterization of PTX-Lips

Two of the most important properties for in vivo integrity and biological fate of nanoparticles (NPs) are particle size and surface charge. In other words, development of carriers with appropriate size and charge plays a crucial role in the field of drug delivery [19, 20]. Several early studies have reported that the cellular uptake efficiency of NPs decreases when increasing the particle size. It is stated that NPs in the range of 100–200 nm have the highest potential to extend circulation time in the bloodstream because they are small enough to avoid mechanical filtration by the spleen, but large enough to avoid selective uptake in the liver. Small size permits NPs passively targeting tumor cells through the enhanced permeability and retention (EPR) effect, improving intracellular accumulation and localization of NPs in tumor area [21, 22]. Another important parameter that controls the stability of NPs in physiological condition is zeta potential. Not only does negative charge in particular improve the physical stability of Lips by preventing them from fusion and aggregation but also the negatively charged NPs are phagocytized significantly less than positive ones [23]. Therefore, particle size and zeta potential are the two key parameters, which have been proven effective for drug delivery applications.

**Figure 2:**
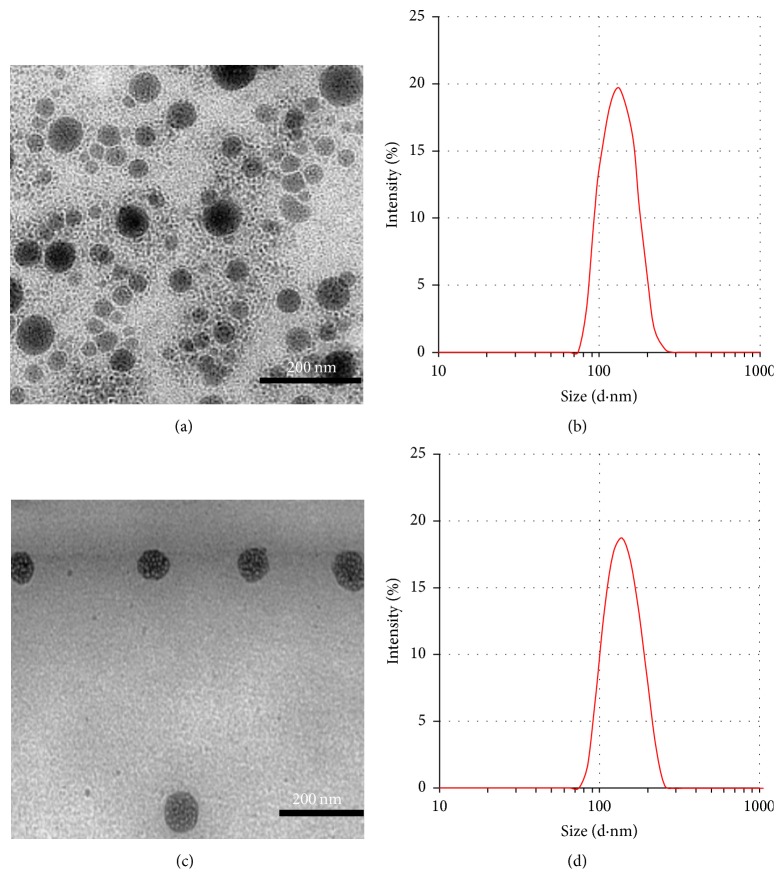
(a, c) TEM image and (b, d) particle size distribution by DLS of Lips and PTX-Lips, respectively.

**Figure 3:**
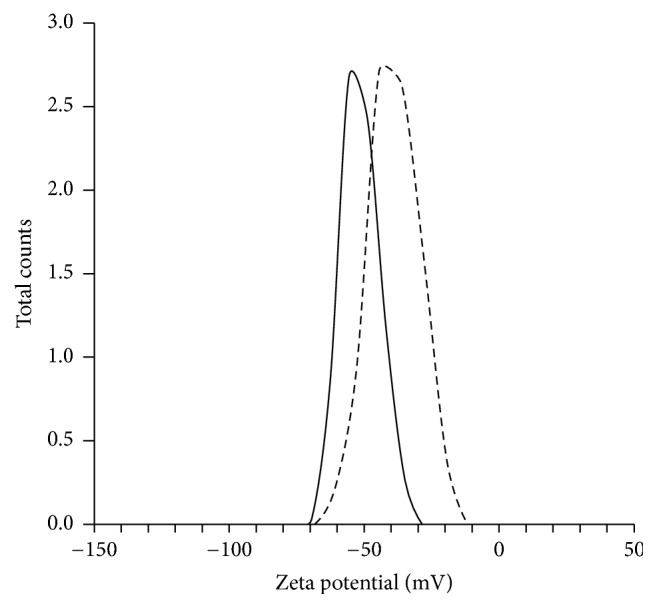
Nanoparticle surface charge via zeta potential of Lips (dashed line) and PTX-Lips (solid line).

As shown in Figures 2(b) and 2(d), the DLS particle size of Lips and PTX-Lips and their population standard deviation were 167 ± 39.1 nm and 131 ± 30.5 nm, whereas the polydispersity index values were 0.286 ± 0.01 and 0.339 ± 0.02, respectively. These results indicated that the particle sizes of Lips and PTX-Lips were not significantly different and their distributions were quite narrow, respectively. Furthermore, the corresponding TEM images (Figures 2(a) and 2(c)) showed that Lips and PTX-Lips were spherical in shape with diameter range of <200 nm and without aggregation or fusion, which were correlated with the values of DLS measurement. Besides particle size, zeta potential values of Lips were approximately −41.7 mV and showed an increase in the surface charge intensity upon inclusion of PTX, −54.3 mV, which may be caused by the PTX interaction with lipid bilayers (Figure 3). Taken together, PTX-Lips might serve as stable spherical nanocarriers with long term circulation in the bloodstream.

### 3.2. Loading and In Vitro Release of PTX

DLE is an important property in drug-loaded nanocarriers and directly affects the therapeutic effect of the system. The higher the encapsulation capacity NPs have, the larger the number of drugs released at the tumor site [24, 25]. In this study, the DLE and DLC of PTX-Lips were found to be 94.5 ± 3.2% and 4.48 ± 0.47%, respectively. In comparison with other studies, Jiang et al. developed novel dual-functional Lips, PTX-loaded 1,2-distearoyl-sn-glycero-3-phosphoethanolamine- (DSPE-) peptide _D_[KLAKLAK]_2_ (KLA) 2,3-dimethylmaleic anhydride (DMA) Lips (DKD/PTX-Lips), to overcome multidrug resistance. The results showed that the DLE of DKD/PTX-Lips was 81.8 ± 0.7% [26]. In another previous study by Zhou et al., the mitochondrial targeting d-a-tocopheryl poly-ethylene glycol 1000 succinate- (TPGS_1000_-) triphenylphosphine (TPP) conjugate (TPGS_1000_–TPP) was synthesized and surface-modified onto PTX-Lips. The targeting PTX-Lips were successfully prepared with DLE of 86.27 ± 3.15% [27]. These results demonstrated that the prepared Lips with the high DLE have the potential to be delivered more efficiently to tumor tissues.

**Figure 4:**
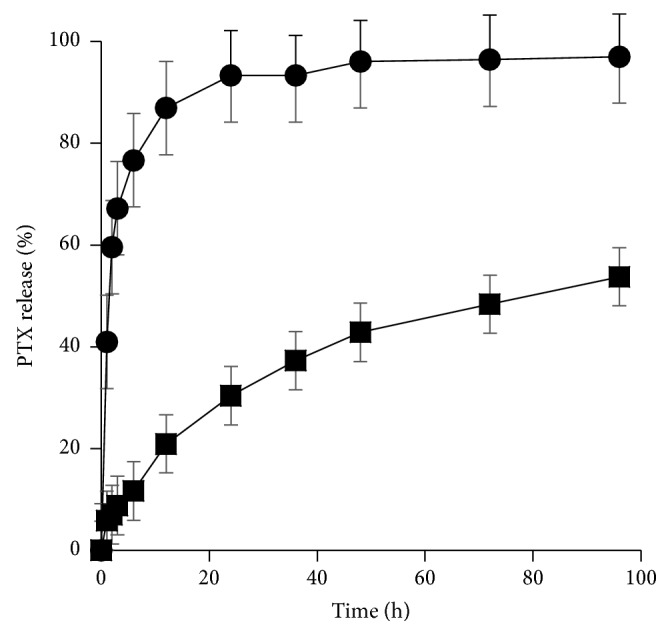
In vitro release profiles of free PTX (circle) and PTX from PTX-Lips (square).

In vitro release profiles of free PTX and PTX from PTX-Lips were performed in order to evaluate the stability and release behavior of PTX-Lips. As shown in Figure 4, the prepared Lips showed a long term stable drug release profile up to 96 h. The cumulative release amount of PTX in first 2 h was around 11% as compared with 60% of free PTX. The initial release of PTX could be explained by the PTX molecules, which were absorbed into the outer phospholipid bilayers of Lips. Zhou et al. reported that the initial release of PTX from the targeting PTX-Lips was less than 30% during the first 2 h [27]. Moreover, total release amount of PTX was 56% after 96 h, compared with 97% of free PTX; in other words, the release behavior of free PTX was significantly faster than PTX in the prepared Lips. This means that the release rate of PTX was dependent on the presence of Lips. Thus, PTX-Lips may serve as stable NPs, therefore increasing drug accumulation into tumor sites.

### 3.3. In Vitro Cytotoxicity

**Figure 5:**
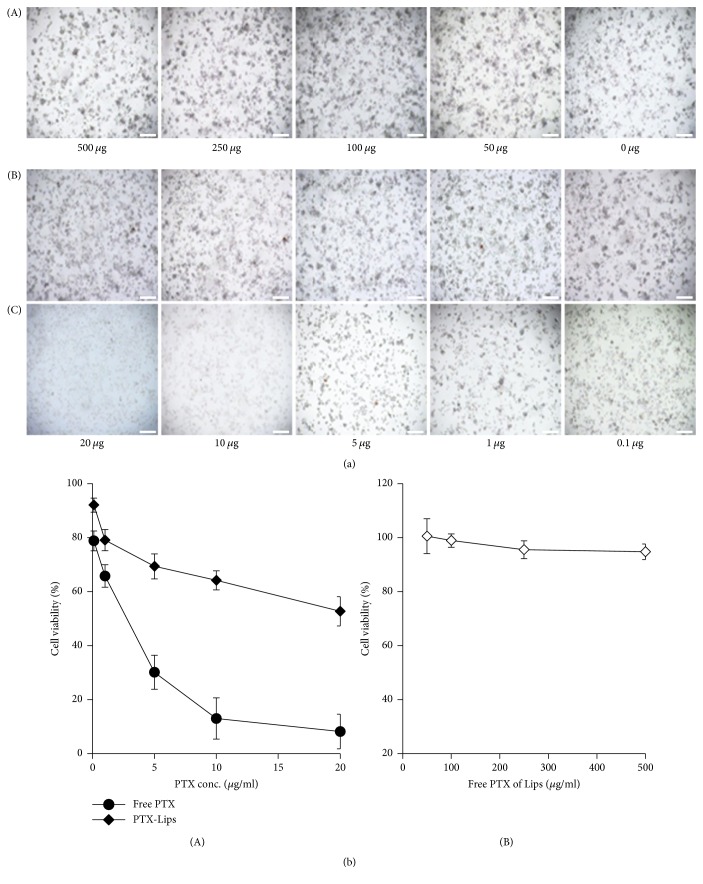
(a) Images of HeLa cells incubated with (A) Lips at different concentrations, (B) PTX-Lips, and (C) free PTX at different PTX doses observed under microscope for 48 h (scale bar = 80 *μ*m) and (b) viability of HeLa cells incubated with (A) free PTX, PTX-Lips at different PTX doses, and (B) free PTX of Lips at different concentrations for 48 h. The cells were exposed to the samples for the indicated times. The data represent the mean values ± the standard deviation (SD) (*n* = 4).

Biocompatibility of a material is an important factor for its success in biomedical applications. In this study, MTT assay was carried out to suggest the biocompatibility of PTX-Lips.  Figure 5 illustrates the inhibitory effects of free PTX, PTX-Lips, and free PTX of Lips on HeLa cells. The blank Lips showed no obvious cytotoxicity towards HeLa cells. Almost 100% of cells were still viable at 500 *μ*g/mL of samples for 2 days, indicating that Lips are biocompatible. On the other hand, the cells growth was significantly inhibited when they were treated with PTX-Lips. A dose-dependent cytotoxicity was observed when individually incubating various doses of free PTX and PTX-Lips with HeLa cells. The majority of cells were killed when they were treated with PTX at concentration of 10 *μ*g/mL for 2 days. The inhibitory effects of Lips and PTX-Lips were consistent with previous researches. It is expected that the toxicity of PTX would be reduced after being encapsulated in Lips. As shown in Figure 5(b)-(A), the percentage of viable cells of PTX-Lips at equivalent PTX concentration of 10 *μ*g/mL was around 64% as compared with that only around 12% of free PTX. These results clearly confirmed that PTX-Lips have the great inhibitory effect against HeLa cells and could be safely used as drug delivery vehicles for in vivo applications.

## 4. Conclusion

Liposomal delivery systems for PTX have been successfully developed by thin film technique. The prepared PTX-Lips were spherical in shape with a diameter around 131 nm, which would be suitable for in vivo drug release. The NPs had DLE and DLC of 94.5 ± 3.2% and 4.48 ± 0.47% and, in addition, the release profile showed sustained release of PTX, respectively. Particularly, it was clear that PTX-Lips could reduce the toxicity of PTX determined by MTT assay. Our results suggest that the Lips made from natural SL have the potential as stable, biocompatible, and efficient PTX delivery systems for the treatment of cancer.
